# “Sometimes the naked taste of potato reminds me of being poor”

**DOI:** 10.3201/eid1506.000000

**Published:** 2009-06

**Authors:** Polyxeni Potter

**Affiliations:** Centers for Disease Control and Prevention, Atlanta, Georgia, USA

**Keywords:** Art science connection, art and medicine, emerging infectious diseases, poverty, nature and rural life, Vincent van Gogh, consumption, tuberculosis, homeless, about the cover

**Figure:**
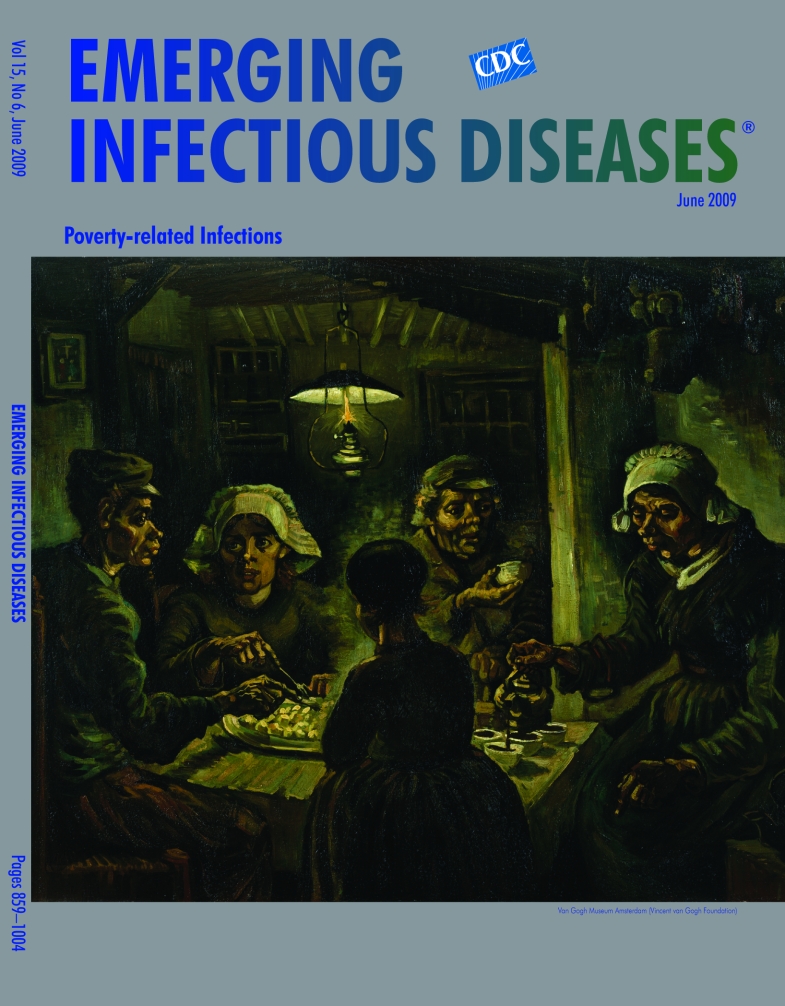
**Vincent van Gogh (1853–1890) The Potato Eaters (1885)** Oil on canvas (81.5 cm × 114.5 cm) Van Gogh Museum, Amsterdam, the Netherlands

―Leonard Nathan

“I want to paint men and women with that something of the eternal which the halo used to symbolize and which we seek to convey by the actual radiance and vibration of our coloring,” wrote Vincent van Gogh in one of his celebrated letters. Hundreds of these were written mostly to his brother Theo, an art dealer in Paris who provided him with financial and emotional support throughout his brief but brilliant career. The letters lay out the artist’s philosophy of life and reveal ample literary inclinations as well as spiritual depth. “Saying a thing well is as interesting and as difficult as painting it,” he wrote.

Van Gogh was born in Zundert, the Netherlands, and was raised in a religious albeit not always harmonious household. “Father cannot understand or sympathize with me …. I too read the Bible … as I read Michelet or Balzac or Eliot … and what Father in his little academic way gleans from it I cannot find in it at all.” Nonetheless, Vincent tried to follow in his father’s evangelical footsteps, but his youthful zeal and empathetic ministry were misinterpreted by the hierarchy of the Dutch Reformed Church. They rejected him, ending his studies in theology and his tenure as missionary to a coal mining community in Belgium.

“Even in that deep misery,” he wrote about his rejection, “I felt my energy revive, and I said to myself, in spite of everything I shall rise again: I will take up my pencil, which I had forsaken in my discouragement, and I will go on with my drawing.” Van Gogh began his artistic career at age 27, while still in Belgium, by painting peasants, whom he perceived as closer to nature than other people, in the manner of his contemporary Jean-François Millet. And with as much zeal as he had pursued his religious mission, he now tried to capture the divine in everyday life.

The Potato Eaters, on this month’s cover, was van Gogh’s first major work. This painting of a family gathered around the table for the evening meal reflected his preoccupation with the plight of the poor, whose lives he had experienced from close up. “The point is,” he wrote to Theo, “I’ve tried to bring out the idea that these people eating potatoes by the light of their lamp have dug the earth with the self-same hands they are now putting into the dish, and it thus suggests *manual labor* and a meal honestly *earned*.”

Depicting night scenes was a creative outlet, a way to test technical innovations and explore the relationship between the cycles of nature and rural life. He drew from the traditions of the 17th-century Dutch masters, particularly Rembrandt, and the Barbizon school landscape painters Charles Daubigny and Jules Dupré. He was also influenced by the impressionists, the pointillists, and Japanese printmakers Hiroshige and Hokusai. Before van Gogh’s untimely death at age 37, these diverse influences had culminated in a unique style, the blend of striking colors and riveting brushstrokes.

“When weavers weave that cloth which I think they call cheviot, or those curious multicolored Scottish tartan fabrics,” van Gogh wrote in reference to the coloring in the Potato Eaters, “then they try, as you know, to get strange broken colors and grays into the cheviot and to get the most vivid colors to balance each other in the multicolored chequered cloth so that instead of the fabric being a jumble, the … pattern looks harmonious from a distance.”

The somber hues and harsh texture of The Potato Eaters went against convention, as did the exaggerated features of the peasants. “I’ve held the threads of this fabric in my hands all winter long and searched for the definitive pattern,” he wrote, “and although it is now a fabric of rough and coarse appearance, the threads have nonetheless been chosen with care and according to certain rules. And it might just turn out to be a *genuine peasant painting. I know that it is*.”

But despite van Gogh’s efforts, this night scene was not well received. It was perceived as not realistic enough, awkward, even technically incorrect. His artistic goals were not understood. “What I try … is not to draw a hand, but the gesture, not a mathematically correct head, but the general expression ….” And his preliminary work was not appreciated. He had done extensive drawings and visited a local family regularly, sketching while they ate. “By continually observing peasant life, at all hours of the day, I have become so involved in it that I rarely think of anything else.”

The life of the poor was also on the literary minds of van Gogh’s day. In 1884–85, naturalist author Émile Zola wrote Germinal, his novel about a coal miners’ strike in northern France in the 1860s. This famous account of poverty and oppression struck a nerve even if it did not end mining strikes or the misery that brought them about. This “wholly different way of life from ours” continued. “According to official statistics just made public for the last six years,” the New York Times reported in 1901, “an average of 150,000 persons have yearly died in France from consumption, while in Paris alone the total for that period has been 83,274 deaths …. All classes have suffered from the disease, but it has been particularly fatal in those sections of the city occupied by working families.”

When he “took up his pencil” against the values of industrial society, van Gogh made no effort to sugarcoat anything. He knew squalor. “Miners, men and women, going to the shaft in the morning through the snow, by a path along a hedge of thorns,” after a day of exhausting labor, they had the color of a “very dusty, unpeeled potato,” their postures showing isolation and resignation.

Poverty, with its attendant malnutrition and crowding, so well captured by van Gogh in The Potato Eaters and by Zola in Germinal, always has been a hotbed of emerging infections. No longer referred to as consumption, TB is still a killer, its rates disproportionately high among the poor. Lice and other pests thrive among the homeless, spreading trench fever and other infections. And proximity to domestic animals and rodents in crowded areas expands the range of influenza, spotted fevers, and plague. But public health efforts to prevent and control the effects of poverty persist. This hope recalls the message of Germinal, “Beneath the blazing sun, in that morning of new growth, the countryside rang with song, as its belly swelled with a black and avenging army of men, germinating slowly in its furrows, growing upwards in readiness for harvests to come, until one day soon their ripening would burst open the earth itself.”
